# Effects and recurrence of proton beam therapy for retinal detachment due to choroidal hemangioma in Sturge–Weber syndrome patients: A case report

**DOI:** 10.1097/MD.0000000000042065

**Published:** 2025-05-30

**Authors:** Kana Tokumo, Yoshiaki Kiuchi, Taro Baba, Naoki Okada, Ayaka Edo, Hiromitsu Onoe, Ariyanie Nurtania, Aisyah Muhlisah, Kazuyuki Hirooka

**Affiliations:** a Department of Ophthalmology and Visual Science, Graduate School of Biomedical Sciences, Hiroshima University, Hiroshima, Japan; b Hiroshima Eye Clinic, Hiroshima, Japan; c Inoue Eye Clinic, Hiroshima, Japan; d RS Mata Makassar, Ministry of Health, Makassar, Indonesia; e RS Andi Sultan Dg Raja Bulukumba, South Sulawesi, Indonesia.

**Keywords:** case report, choroidal hemangioma, proton beam therapy, retinal detachment, Sturge–Weber syndrome

## Abstract

**Rationale:**

The aim of this study was to evaluate the clinical outcomes and recurrence patterns of proton beam therapy for choroidal hemangioma–associated retinal detachment in patients with Sturge–Weber syndrome, addressing the limitations of conventional treatments and further elucidating posttreatment recurrence patterns.

**Patient concerns:**

Three patients with Sturge–Weber syndrome presented with retinal detachment secondary to diffuse choroidal hemangioma, refractory to standard therapies.

**Diagnoses:**

All patients were diagnosed with choroidal hemangioma complicated by subretinal fluid accumulation and retinal detachment in the context of Sturge–Weber syndrome.

**Interventions:**

Each patient underwent targeted proton beam therapy directed at the choroidal hemangioma.

**Outcomes:**

In all 3 cases, subretinal fluid gradually decreased and ultimately resolved. One patient experienced initial tumor thinning followed by a subsequent increase in hemangioma thickness accompanied by reappearance of subretinal fluid, indicating recurrence.

**Lessons:**

Proton beam therapy appears to be an effective treatment modality for retinal detachment associated with choroidal hemangioma in Sturge–Weber syndrome. However, an increase in hemangioma thickness after initial response signals a risk of recurrence; therefore, regular optical coherence tomography and B-mode ophthalmic ultrasound examinations are essential for early detection and timely intervention.

## 1. Introduction

Sturge–Weber syndrome (SWS) is associated with diffuse choroidal hemangiomas in 20% to 70% of patients.^[1]^ SWS is a congenital, nonhereditary disorder that is characterized by facial port-wine strain. It occurs in both male and female newborns, with an incidence of approximately 1 in 20,000 to 50,000 live births.^[2]^ SWS is related to a somatic mutation in the GNAQ gene.^[3]^ In patients with SWS, brain involvement typically presents in infancy with seizures, strokes, stroke-like episodes, and a range of neurologic impairments.^[4]^ Standard treatment includes laser therapy for birthmarks and the use of anticonvulsants. Increased choroidal thickness in SWS patients may lead to severe retinal complications, such as subretinal fluid, retinal degeneration, retinal detachment, tortuous retinal vessels, and optic disc coloboma.^[5,6]^ If the patient is asymptomatic, no treatment is necessary.^[5]^ Circumscribed choroidal hemangiomas rarely increase in size and may remain quiescent for months or years.^[7]^ The onset of exudative retinal detachment and the duration of persistent exudative changes significantly affect the visual prognosis of SWS patients.^[8]^ If untreated, retinal detachment can lead to neovascular glaucoma, a refractory form of glaucoma.^[1]^

Retinal detachment due to choroidal hemangioma can be managed with various therapies, including internal beta-blockers, low-power infrared laser therapy, photodynamic therapy (PDT), and radiation therapy, such as external beam radiation therapy, proton beam therapy, stereotactic radiotherapy, and plaque radiotherapy.^[9,10]^ Unfortunately, the effectiveness of these treatments is limited, and many patients require a combination of treatments.^[10–12]^

We observed retinal detachment in 3 young patients with SWS who underwent proton beam therapy. In these patients, retinal detachment improved, and choroidal hemangiomas shrank and remained stable over a long period. Retinal detachment recurred in 1 eye after proton beam therapy. Although several reports on proton beam treatment for hemangiomas due to SWS have been published,^[5,6,13,14]^ recurrent retinal detachment has not been reported. We also compared patients without recurrence to provide a comprehensive perspective.

## 2. Materials and methods

Three patients with SWS were treated for choroidal detachment and secondary glaucoma at Hiroshima University Hospital, Hiroshima, Japan. Verbal consent was obtained from the families of the affected children in this case report.

**Figure 1.**
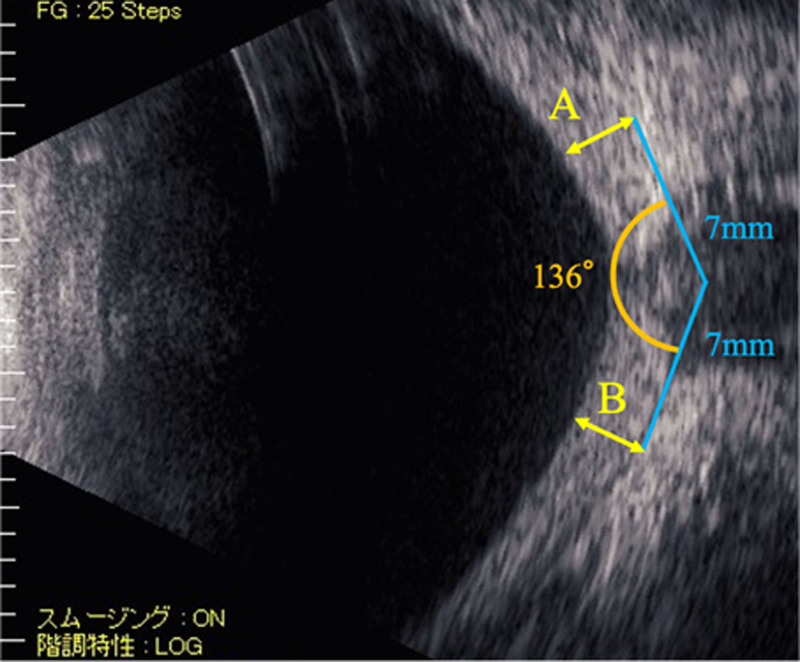
Changes in the size of the choroidal hemangioma were assessed by comparing the sum of the height of the choroidal hemangioma (A + B) at 7 mm from the optic nerve. To maintain accuracy in the evaluation, the angle between the 2 points connected from the optic nerve was set to 136° in all cases.

B-mode ophthalmic ultrasonography was used to measure the thickness of the choroidal hemangioma at 2 points: 7 mm from the optic nerve on the temporal and nasal sides (or superiorly and inferiorly), with an angle of 136° between the 2 points. This distance was chosen because many cases had a maximum height close to this point. After various angles were tested across all the cases to establish a universally applicable angle, this measurement method was chosen for its accuracy. The sum of these measurements provided the thickness of the choroidal hemangioma. In cases where the border between the sclera and choroid was indistinct, the sclera was included to assess the height of the choroidal hemangioma. An example of this measurement is shown in Figure 1.

## 3. Case presentation

### 3.1. Patient 1

A male patient was diagnosed with SWS due to hemangiomas affecting the regions of the first and second branches of the trigeminal nerve on both sides of his face since his birth in 2001. He also developed secondary glaucoma in both eyes, with the right eye undergoing 4 trabeculotomies and 1 Baerveldt implant surgery by 2014. The left eye underwent 3 trabeculotomies. Choroidal hemangiomas were observed in both eyes via B-mode ophthalmic ultrasound, with the retina exhibiting a reddish appearance resembling that of tomato ketchup.

In December 2014, the right eye developed retinal detachment. Intravitreal injection of vascular endothelial growth factor (VEGF) and PDT were ineffective. In July 2016, proton beam therapy (19.8 gray equivalents [Gy]/11 sessions) was performed, and the disappearance of the subretinal fluid was confirmed in December 2016. In April 2019, retinal detachment recurred in the right eye but spontaneously resolved after 5 months.

Retinal detachment of the left eye occurred in April 2019. In February 2020, proton beam therapy was planned but postponed because the coronavirus disease 2019 pandemic prevented hospital visits. Consequently, scleral fenestration surgery was performed in the left eye, but its effectiveness was limited, and retinal detachment persisted. In August 2021, proton beam therapy (19.8 Gy/11 sessions) was administered, which resulted in the disappearance of retinal detachment by November 2021.

**Figure 2.**
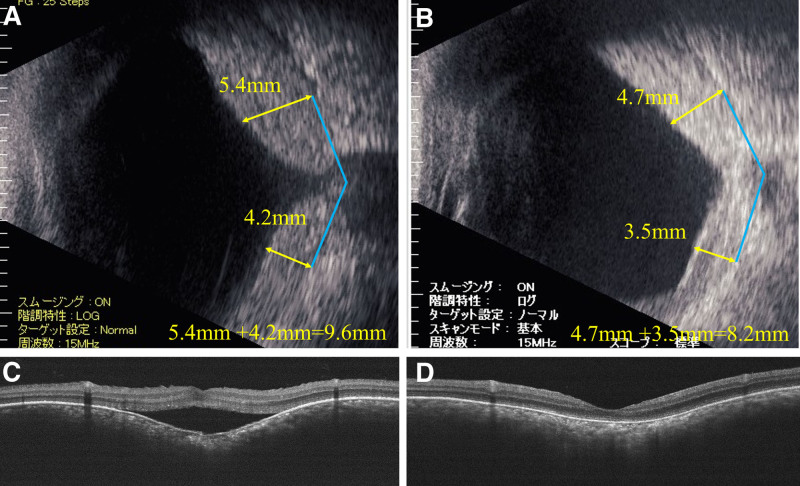
(A) B-mode ophthalmic ultrasound before proton beam therapy in the right eye. The height of the hemangioma was 9.6 mm (5.4 + 4.2 mm). (B) B-mode ophthalmic ultrasound 45 mo after proton beam therapy in the right eye. The height of the hemangioma was 5.7 mm (3.4 + 2.3 mm). (C) OCT before proton beam therapy. Mild retinal detachment was observed only in the macula. (D) OCT 45 mo after proton beam therapy. Retinal detachment improved. OCT = optical coherence tomography.

**Figure 3.**
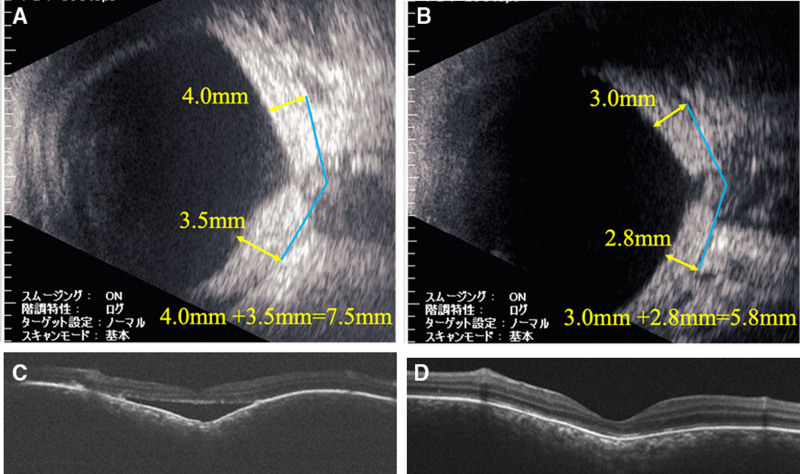
(A) B-mode ophthalmic ultrasound before proton beam therapy in the left eye. The height of the hemangioma was 7.5 mm (4.0 + 3.5 mm). (B) B-mode ophthalmic ultrasound 7 mo after proton beam therapy in the left eye. The height of the hemangioma was 5.8 mm (3.0 + 2.8 mm). (C) OCT before proton beam therapy. Mild retinal detachment was observed only in the macula. (D) OCT 7 mo after proton beam therapy. Retinal detachment improved. OCT = optical coherence tomography.

**Figure 4.**
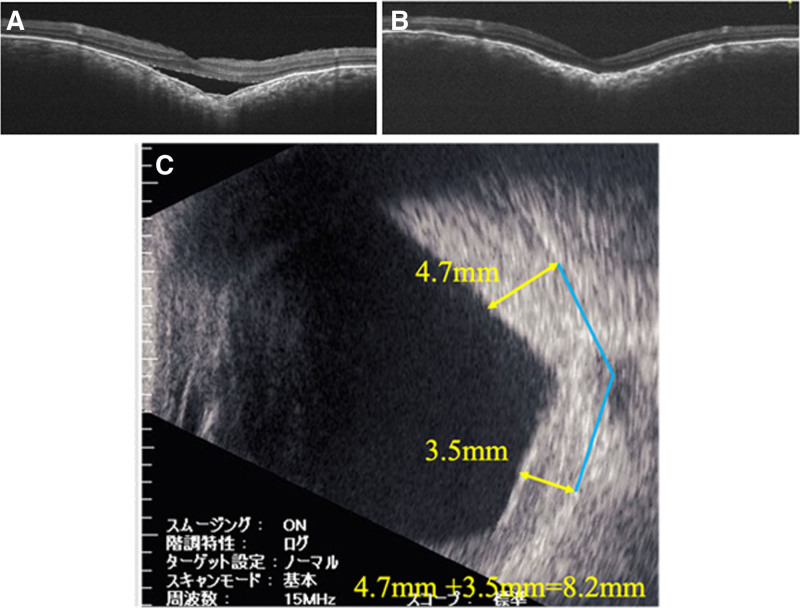
(A) OCT when retinal detachment recurred in the right eye in April 2019. (B) OCT when retinal detachment improved in the right eye in May 2020. (C) B-mode ophthalmic ultrasound in May 2020 after recurrent retinal detachment and improvement in the right eye. The height of the hemangioma was 8.2 mm (4.7 + 3.5 mm). OCT = optical coherence tomography.

B-mode ophthalmic ultrasound and optical coherence tomography (OCT) images before and after proton beam therapy for the right eye are shown in Figure 2, and those for the left eye are shown in Figure 3. The sum of the height of the choroidal hemangioma at 7 mm from the optic nerve decreased from 9.6 mm (5.4 + 4.2 mm) to 5.7 mm (3.4 + 2.3 mm) in the right eye over 45 months and from 7.5 mm (4.0 + 3.5 mm) to 5.8 mm (3.0 + 2.8 mm) in the left eye over 7 months posttherapy. After recurrence of retinal detachment in the right eye, the thickness of the hemangioma on B-mode ophthalmic ultrasound increased to 8.2 mm (4.7 + 3.5 mm), as shown in Figure 4. The choroidal hemangiomas in both eyes thinned after proton beam therapy. However, in the right eye, where retinal detachment recurred, the choroidal hemangioma thickened again.

### 3.2. Patient 2

Since birth in 2004, a female patient has had a hemangioma affecting the first and second branches of the trigeminal nerve on the left side of her face, along with a diagnosis of SWS. She presented with corneal opacity and bull’s-eye appearance in her left eye, leading to a diagnosis of secondary glaucoma. The patient underwent trabeculotomy 3 times by 2008. The fundus of her left eye was reddish, with a tomato ketchup appearance.

**Figure 5.**
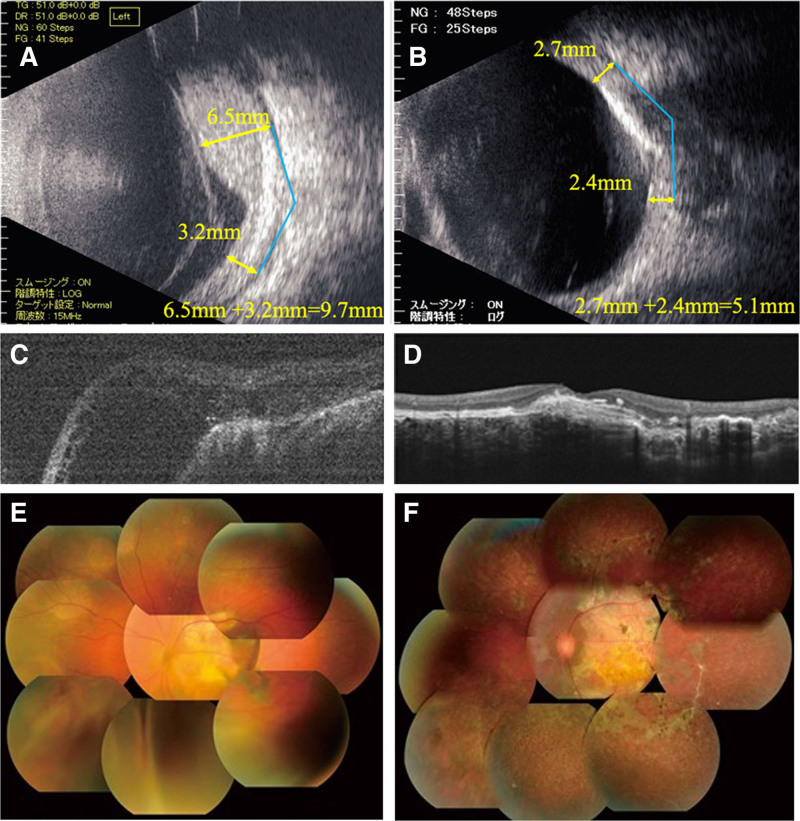
(A) B-mode ophthalmic ultrasound before proton beam therapy. The height of the hemangioma was 9.7 mm (6.5 + 3.2 mm). (B) B-mode ophthalmic ultrasound performed 49 mo after proton beam therapy. The height of the hemangioma was 5.1 mm (2.7 + 2.4 mm). (C) OCT before proton beam therapy. Retinal detachment and macular edema were observed. (D) OCT was performed 49 mo after proton beam therapy. Retinal thinning and partial choroidal fibrosis were observed. (E) Fundus photograph obtained before proton beam therapy. Severe retinal detachment was observed inferiorly. (F) Fundus photograph taken 49 mo after proton beam therapy. The retina was found to be atrophied. OCT = optical coherence tomography.

In March 2011, diffuse exudative retinal detachment associated with a choroidal hemangioma developed in the left eye. Treatments included internal beta-blocker use, PDT, anti-VEGF injections, and choroidal cryocoagulation; however, the choroidal detachment did not improve. Therefore, in June 2014, she underwent proton beam therapy (20 Gy/10 sessions) in the left eye. The choroidal detachment gradually decreased and was completely resolved by August 2014. The choroidal hemangioma also thinned and remained stable. B-mode ultrasound, fundus photographs, and OCT images before and 49 months after proton beam therapy are shown in Figure 5. The sum of the height of the choroidal hemangioma at 7 mm from the optic nerve decreased from 9.7 mm (6.5 + 3.2 mm) to 5.1 mm (2.7 + 2.4 mm) following proton beam therapy.

### 3.3. Patient 3

A male patient had a hemangioma affecting the first and second branches of the trigeminal nerve on the left side of his face since birth in 2016. In 2019, B-mode ophthalmic ultrasound results were normal, but the left fundus exhibited a reddish appearance resembling that of a tomato ketchup.

**Figure 6.**
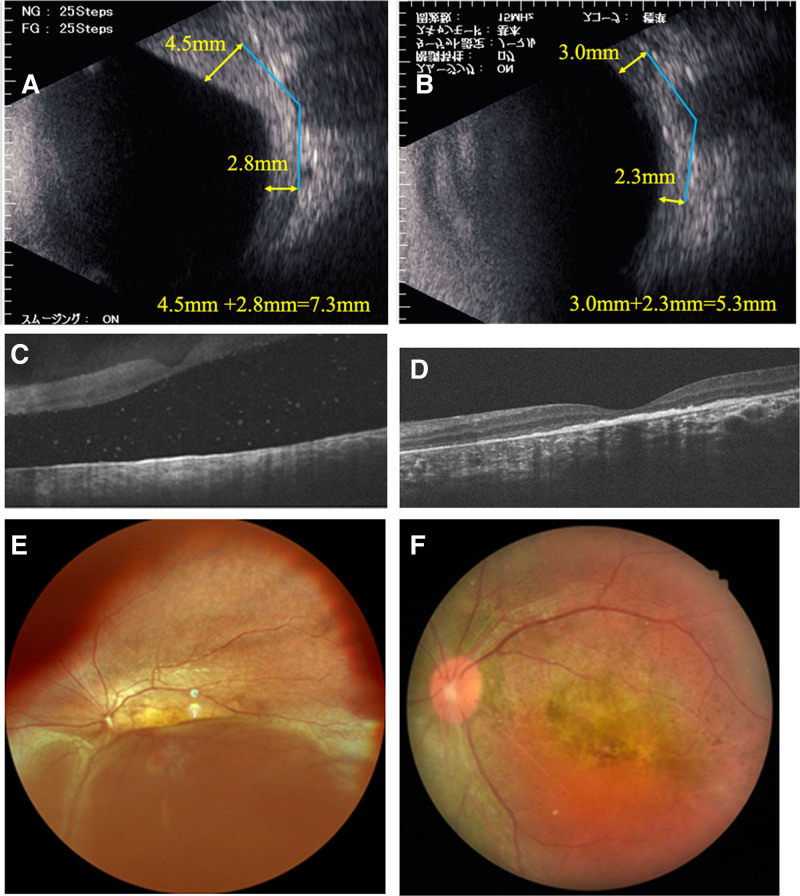
(A) B-mode ophthalmic ultrasound before proton beam therapy. The height of the hemangioma was 7.3 mm (4.5 + 2.8 mm). (B) B-mode ophthalmic ultrasound performed 10 mo after proton beam therapy. The height of the hemangioma was 5.3 mm (3.0 + 2.3 mm). (C) OCT before proton beam therapy. The macular retina was completely detached. (D) OCT 10 mo after proton beam therapy. Retinal detachment improved. (E) Fundus photograph obtained before proton beam therapy. Severe retinal detachment was observed inferiorly. (F) Fundus photograph taken 10 mo after proton beam therapy. The retina was found to be atrophied. OCT = optical coherence tomography.

In July 2021, B-mode ophthalmic ultrasound revealed a choroidal hemangioma, and OCT revealed retinal detachment in the left eye. PDT was performed in January 2022 but did not improve the patient’s condition; instead, the choroidal detachment worsened. Consequently, in May 2022, proton beam therapy was administered (19.8 Gy/11 sessions). Following proton beam therapy, the choroidal detachment gradually decreased and was completely resolved by December 2022. The choroidal hemangioma also thinned and remained stable. B-mode ophthalmic ultrasound, fundus photographs, and OCT images obtained before and 10 months after proton beam therapy are shown in Figure 6. The sum of the height of the choroidal hemangioma at 7 mm from the optic nerve decreased from 7.3 mm (4.5 + 2.8 mm) to 5.3 mm (3.0 + 2.3 mm) following proton beam therapy.

## 4. Discussion

Proton beam therapy is effective in treating diffuse retinal detachment caused by choroidal hemangiomas. In these patients, not only did the choroidal detachment improve, but the choroidal hemangiomas also decreased in size. Even when the size of the retinal vascular tumor had decreased following proton beam therapy, an increased thickness of the choroidal hemangioma was observed in cases exhibiting recurrent retinal detachment.

Indocyanine green angiography can depict tumors more clearly than fluorescein angiography can.^[15]^ In the early stages, reticulated intertumoral vessels are observed, and strong hyperfluorescence is observed over time. In later stages, the fluorescent dye was washed out. These findings suggest that exudative retinal detachment occurs due to excessive exudate from the hemangioma and retinal pigment epithelial damage caused by choroidal hemangioma.

Previously, laser photocoagulation was used to treat diffuse retinal detachment due to choroidal hemangioma.^[16,17]^ However, recurrence is frequent, and the visual prognosis is often poor. There are several other treatments for choroidal hemangiomas, such as PDT, intravitreal injections of anti-VEGF agents, internal beta-blocker use, low-power infrared laser therapy, and radiation therapy.^[9,10]^

PDT selectively targets the choroidal vasculature with verteporfin, which primarily results in vascular occlusion and is relatively noninvasive.^[18]^ Intravitreal injections of anti-VEGF agents inhibit the formation of abnormal vascular networks and reduce vascular permeability by suppressing VEGF.^[11,19]^ The nonselective sympathetic beta-blocker propranolol promotes vasoconstriction in hemangiomas by inhibiting nitric oxide release and angiogenic signals, including VEGF and basic fibroblast growth factor, and promotes the apoptosis of proliferating vascular endothelial cells.^[20]^ In low-power infrared laser therapy, the heating effect results in tumor necrosis, which generally develops within a few days of treatment.^[7]^ Radiation therapy is believed to reduce the size of vascular hemangiomas by inhibiting the formation of thrombi that occlude tumor vessels and the proliferation of endothelial cells within the tumor, which in turn reduces exudate and improves retinal detachment.^[17,21]^

Radiation therapy can be broadly divided into external beam radiation therapy and internal radiation therapy (brachytherapy).^[1]^ In external beam radiation therapy, high-energy radiation—such as X-rays, gamma rays, or charged particles (e.g., protons or carbon ions)—is administered from a source positioned outside the patient’s body. In contrast, brachytherapy involves placing radioactive sources within or very close to the tumor, thereby delivering a concentrated dose of radiation to malignant cells while minimizing exposure to surrounding healthy tissues. Ophthalmic plaque therapy, which employs radioisotopes such as ruthenium-106 or iodine-125, is one form of brachytherapy used specifically for ocular tumors. In this technique, a small radioactive “plaque” is temporarily affixed to the outer surface of the eye (the sclera), allowing targeted irradiation of the tumor and reducing collateral damage to critical structures such as the retina and optic nerve.^[22,23]^

Proton beams are a form of radiation. The advantage of proton beam irradiation is that a homogeneous dose can be delivered to the target while sparing healthy tissue surrounding the tumor. Charged protons have a physical property known as the Bragg peak.^[13,24]^ After the maximum dose is administered at a certain depth, the relative dose decreases steeply. By adjusting the depth and width of the peak according to the depth and size of the lesion, the radiation can be concentrated on the lesion, and the dose disappears behind it. The proton was not irradiated distally to the target. In X-ray-based radiation therapy regimens, a buildup effect occurs.^[24]^ After delivery of the peak dose, the deeper the rays penetrate the body, the more gradual the decrease in the relative dose becomes as the rays pass through the body. While radiotherapy may destroy hemangiomas, late complications of proton beam therapy, including radiation maculopathy and optic neuropathy, may limit its therapeutic benefit. The disadvantages of proton therapy are that it can be performed only in a limited number of facilities and that access is difficult owing to the remoteness of these facilities. In such cases, school-aged children typically wait until the summer break to receive treatment. Owing to the spread of the coronavirus disease 2019 (COVID-19) virus, restrictions on long-distance travel resulted in patients waiting for approximately 6 months to receive treatment.

A reduction in choroidal hemangiomas after proton beam radiation therapy has been reported. Franco reported that the maximum tumor thickness decreased after proton beam therapy from a median of 4.6 mm (range 1.7–5.8) to a median of 3.0 mm (range 1–3.7).^[25]^ In the 2 cases reported by Yonekawa, the height of the tumors decreased from 5.4 to 3.0 mm and from 5.8 to 2.7 mm.^[13]^ The tumor thickness also decreased in all of our patients.

The irradiated proton doses varied among the studies, but some cases were influenced by the historical background of the time. Franco et al^[25]^ reported that 9 patients received 20 Gy in 10 fractions, whereas 2 patients were treated with an earlier protocol of 15 Gy in 4 fractions. Zografos et al^[6]^ reported that patients received 27.3, 22.7, and 16.4–18.2 Gy in 4 fractions of proton therapy. Höcht et al^[5]^ used 22.5 Gy in 6 fractions, and Chan et al^[14]^ reported 15–30 Gy in 4 fractions. Yonekawa et al^[13]^ used a dose of 20 Gy in 10 fractions. Retinal detachment was resolved in all patients, and there were no cases of recurrent subretinal fluid. Among our patients, there was a recurrence of retinal detachment in 1 patient, in whom the hemangioma thinned only slightly and remained thick. It is believed that the hemangioma remained highly active.

In a patient with recurrent retinal detachment, the choroidal hemangioma temporarily thinned after proton beam therapy but then thickened again. This recurrence could be due to increased activity of the choroidal hemangioma, causing thickening and retinal detachment. Proton beam therapy should be used to irradiate hemangiomas until they become nonfunctional. The radiation dose in this case was 19.8 Gy in 11 fractions, which is comparable to other reports. This result suggests that the therapeutic effects of proton irradiation may vary from 1 individual to another, even with the same radiation dose. Careful consideration of the irradiation dose is necessary because complications such as radiation retinopathy increase as the dose increases. In particular, when irradiation is administered to previously treated areas, late side effects are more likely to occur than with the first irradiation, and there is a greater possibility that the side effects will be more prominent than the effects of radiation.^[26]^ Our patient experienced spontaneous resolution of subretinal fluid after recurrence, but treatments for patients whose symptoms do not resolve spontaneously need to be considered.

After proton beam therapy, B-mode ophthalmic ultrasound was performed irregularly following thinning of the choroidal hemangioma and improvement of retinal detachment. Therefore, the last B-mode ophthalmic ultrasound before recurrence is shown in Figure 2B and was taken 11 months before recurrence. Regular B-mode ophthalmic ultrasound monitoring may have detected enlargement of the choroidal hemangioma, and the recurrence of retinal detachment may have been predicted.

Proton beam radiation has shown promising results in such cases. However, the collateral damage caused by these treatments, combined with the uneven delivery of radiation to the tumor, has driven the search for more localized methods of therapy. Several reports have documented improvements in choroidal hemangioma and retinal detachment with brachytherapy, and we remain hopeful about the potential of brachytherapy for treating these conditions.^[23,27]^

The main limitation of this report is that the hemangioma was evaluated by B-mode ophthalmic ultrasound, which is not reproducible at the same site, and was evaluated by summing the heights of the sites 7 mm from the optic nerve. Additionally, the boundary between the sclera and choroid may not be distinguished; in these cases, the tumor size could be more accurately assessed by magnetic resonance imaging (MRI). In terms of accessibility and examination costs, OCT and B-mode ophthalmic ultrasound are generally easier to perform than MRI. OCT can measure choroidal thickness more accurately than can B-mode ophthalmic ultrasound.^[28]^ Hence, an accurate evaluation with OCT is crucial; however, in the present case, the choroidal thickness exceeded the device’s measurable range, making OCT assessment difficult. Consequently, we performed the evaluation using B-mode ophthalmic ultrasound. Therefore, OCT and B-mode ophthalmic ultrasound are preferable if frequent evaluations are needed. However, owing to its superior accuracy, MRI is preferred for periodic evaluation, and more frequent OCT and B-mode ophthalmic ultrasound examinations are necessary.

Furthermore, several additional limitations merit consideration. First, this study is based on a limited sample size, potentially a single case, thereby restricting the generalizability of the findings. Second, measurements obtained via B-mode ophthalmic ultrasound are operator dependent, introducing the possibility of both interobserver and intraobserver variability. Third, inherent measurement errors and calibration issues associated with the ultrasound device may further compromise the accuracy and reproducibility of the results. Fourth, limitations in imaging techniques could affect the reliability of long-term monitoring and follow-up evaluations, particularly in the assessment of disease progression or therapeutic response. Finally, patient-specific factors such as cooperation, ocular media opacities, and individual anatomical variations might also influence imaging quality and thereby affect the overall evaluation.

In conclusion, proton therapy is effective in the treatment of retinal detachment due to choroidal hemangiomas associated with SWS. In cases where the choroidal hemangioma thinned after proton beam therapy but later thickened again, retinal detachment recurred. Owing to the high likelihood of recurrence of retinal detachment and the emergence of other complications in the future, careful long-term monitoring via OCT and B-mode ophthalmic ultrasound is necessary. By detecting an increase in the thickness of the choroidal hemangioma with OCT and B-mode ophthalmic ultrasound, the recurrence of retinal detachment may be anticipated.

## Acknowledgments

We thank American Journal Experts (https://www.aje.com) for editing a draft of this manuscript.

## Author contributions

**Conceptualization:** Kana Tokumo, Yoshiaki Kiuchi, Taro Baba, Naoki Okada, Ayaka Edo, Hiromitsu Onoe, Kazuyuki Hirooka.

**Data curation:** Ayaka Edo.

**Investigation:** Kazuyuki Hirooka.

**Methodology:** Kana Tokumo, Yoshiaki Kiuchi, Kazuyuki Hirooka.

**Project administration:** Kana Tokumo.

**Resources:** Kana Tokumo, Ariyanie Nurtania, Aisyah Muhlisah.

**Supervision:** Yoshiaki Kiuchi, Kazuyuki Hirooka.

**Validation:** Taro Baba, Naoki Okada, Hiromitsu Onoe.

**Visualization:** Taro Baba.

**Writing—original draft:** Kana Tokumo.
